# *rac*-2-Iodo-3,4-dihydro­naphthalen-1(2*H*)-one

**DOI:** 10.1107/S1600536809049095

**Published:** 2009-11-21

**Authors:** Abdul Rauf Raza, M. Nawaz Tahir, Ayesha Sultan, Muhammad Danish, Muhammad Sohail

**Affiliations:** a Department of Chemistry, University of Sargodha, Sargodha, Pakistan; b Department of Physics, University of Sargodha, Sargodha, Pakistan

## Abstract

In the title compound, C_10_H_9_IO, the asymmetric unit contains two mol­ecules, in which the iodo-bearing six-membered rings adopt envelope conformations [displacements of the flap atoms = 0.419 (3) and 0.431 (3) Å]. In both mol­ecules, the I atoms are disordered over two set of sites in 0.54 (4):0.46 (4) and 0.71 (3):0.29 (3) ratios. In the crystal, the packing features a weak C—H⋯π inter­action.

## Related literature

For a related structure, see: Haddad (1986 ▶).
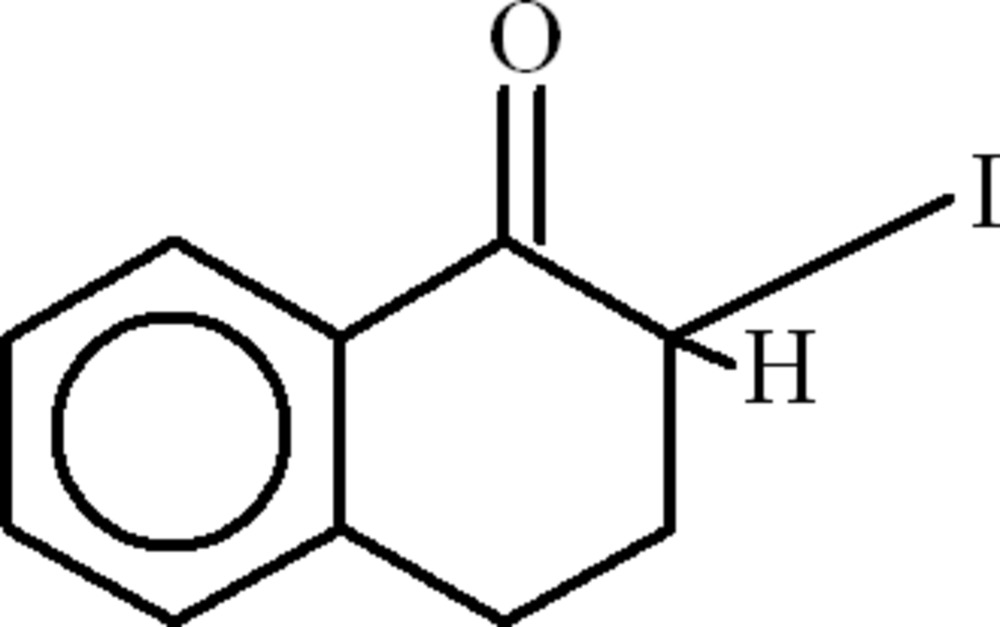


## Experimental

### 

#### Crystal data

C_10_H_9_IO
*M* *_r_* = 272.07
Monoclinic, 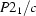

*a* = 6.115 (5) Å
*b* = 19.658 (4) Å
*c* = 15.896 (5) Å
β = 90.551 (5)°
*V* = 1910.7 (17) Å^3^
*Z* = 8
Mo *K*α radiation
μ = 3.31 mm^−1^
*T* = 296 K
0.28 × 0.20 × 0.18 mm

#### Data collection

Bruker Kappa APEXII CCD diffractometer
Absorption correction: multi-scan (*SADABS*; Bruker, 2005 ▶) *T*_min_ = 0.685, *T*_max_ = 0.717
19043 measured reflections
4397 independent reflections
3632 reflections with *I* > 2σ(*I*)
*R*_int_ = 0.026

#### Refinement

*R*[*F* ^2^ > 2σ(*F* ^2^)] = 0.024
*wR*(*F* ^2^) = 0.059
*S* = 1.07
4397 reflections
238 parameters
H-atom parameters constrained
Δρ_max_ = 0.39 e Å^−3^
Δρ_min_ = −0.50 e Å^−3^

### 

Data collection: *APEX2* (Bruker, 2007 ▶); cell refinement: *SAINT* (Bruker, 2007 ▶); data reduction: *SAINT*; program(s) used to solve structure: *SHELXS97* (Sheldrick, 2008 ▶); program(s) used to refine structure: *SHELXL97* (Sheldrick, 2008 ▶); molecular graphics: *ORTEP-3* (Farrugia, 1997 ▶) and *PLATON* (Spek, 2009 ▶); software used to prepare material for publication: *WinGX* (Farrugia, 1999 ▶) and *PLATON*.

## Supplementary Material

Crystal structure: contains datablocks global, I. DOI: 10.1107/S1600536809049095/hb5236sup1.cif

Structure factors: contains datablocks I. DOI: 10.1107/S1600536809049095/hb5236Isup2.hkl

Additional supplementary materials:  crystallographic information; 3D view; checkCIF report

## Figures and Tables

**Table 1:** Hydrogen-bond geometry (Å, °)

*D*—H⋯*A*	*D*—H	H⋯*A*	*D*⋯*A*	*D*—H⋯*A*
C5—H5⋯*Cg*3	0.93	2.95	3.700 (4)	139

*Cg*3 is the centroid of the C11–C16 ring.

**Fig. 1.**
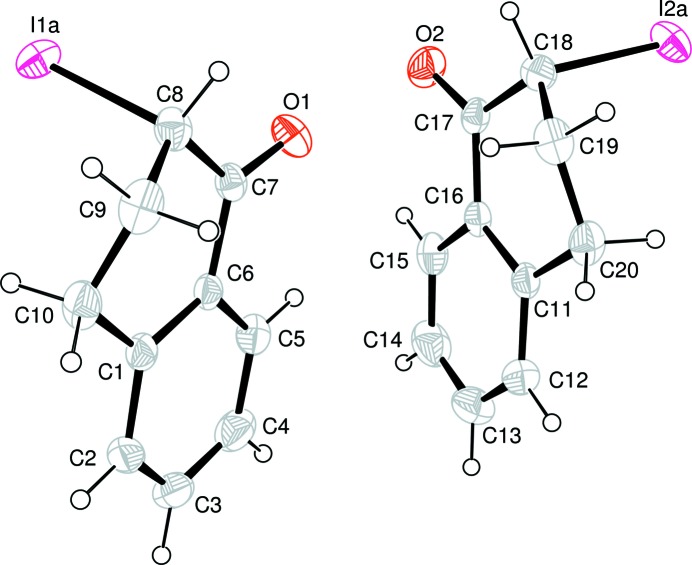
View of (I) with the I atoms having greater occupancies. The displacement ellipsoids are drawn at the 30% probability level.

**Fig. 2.**
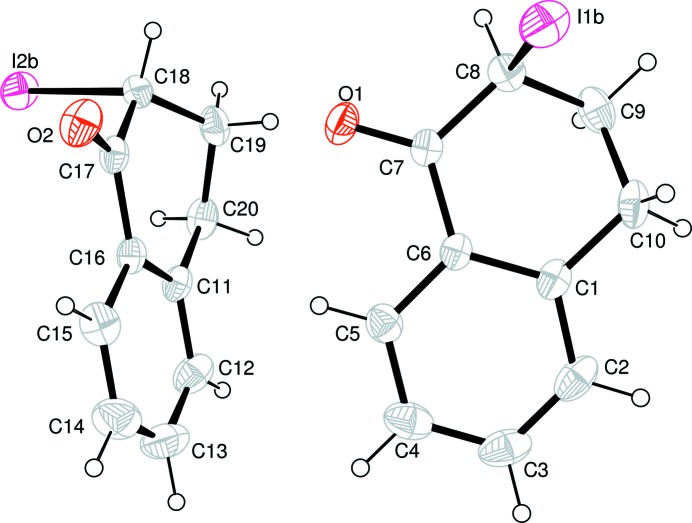
View of (I) with the I atoms having lesser occupancies. The displacement ellipsoids are drawn at the 30% probability level.

## supplementary crystallographic information

### Comment

The title compound (I, Fig. 1) is an intermediate for the total synthesis of steroidal hormones. The ctystal structures of (II) 2,2-Dibromo-3,4-dihydro-1(2*H*)-naphthalenone (Haddad, 1986) has been published, which seems relavent to (I).

The asymmetric unit of title compound consists of two individual molecules which are clearly racemate. In the molecule having (*S*)-configuration, the I-atom containing ring A (C1/C6/C7—C10) is twisted with maximum puckering amplitude Q_T_ = 0.431 (3) Å, whereas in (*R*)-configuration the puckering parameter is Q_T_ = 0.419 (3) Å. In two molecules the groups of benzene rings along with two adjacent C-atoms, C (C1—C6/C7/C10) and D (C11—C16/C17/C20) are planar with maximum r. m. s. deviations of 0.0114 and 0.0280 Å respectively, from the respective mean square planes. The dihedral angle between C/D is 66.83 (7) Å. In the first molecule the I-atom is disordered over two set of sites having occupancy ratio of 0.54 (4):0.46 (4). Similarly in the other molecule the I-atom is disordered over two set of sites having occupancy ratio of 0.71 (3):0.29 (3). The molecules are stabilized due to C–H···π interactions (Table 1).

### Experimental

A solution of I_2_ (7.75 g, 30.5 mmol) in CHCl_3_ was added as drops to a solution of 1-tetralone (2.198 g, 15.2 mmol) in acetic acid (9.156 g, 0.1526 mol) and refluxed for 28 h. The H_2_O (30 ml) was added for partitioning. The reaction mixture was extracted with CHCl_3_ (3 × 15 ml). The combined organic layer was concentrated *in vacuo*, the crude was dissolved in ethyl acetate, washed with 5% Na_2_S_2_O_3_ (2 × 15 ml), dried over anhydrous Na_2_SO_4_, filtered, boiled with charcoal, concentrated under reduce pressure and allowed for crystallization, which afforded colourless prisms (89%) of (I).

### Refinement

The other H-atoms were positioned geometrically (C–H = 0.93–0.98 Å) and refined as riding with *U*_iso_(H) = 1.2*U*_eq_(C).

### Crystal data

**Table:**

C_10_H_9_IO	*F*(000) = 1040
*M**_r_* = 272.07	*D*_x_ = 1.892 Mg m^−^^3^
Monoclinic, *P*2_1_/*c*	Mo *K*α radiation, λ = 0.71073 Å
Hall symbol: -P 2ybc	Cell parameters from 4397 reflections
*a* = 6.115 (5) Å	θ = 2.4–27.8°
*b* = 19.658 (4) Å	µ = 3.31 mm^−^^1^
*c* = 15.896 (5) Å	*T* = 296 K
β = 90.551 (5)°	Prism, colourless
*V* = 1910.7 (17) Å^3^	0.28 × 0.20 × 0.18 mm
*Z* = 8	

### Data collection

**Table:**

Bruker Kappa APEXII CCD diffractometer	4397 independent reflections
Radiation source: fine-focus sealed tube	3632 reflections with *I* > 2σ(*I*)
graphite	*R*_int_ = 0.026
Detector resolution: 7.50 pixels mm^-1^	θ_max_ = 27.8°, θ_min_ = 2.4°
ω scans	*h* = −8→7
Absorption correction: multi-scan (*SADABS*; Bruker, 2005)	*k* = −25→24
*T*_min_ = 0.685, *T*_max_ = 0.717	*l* = −20→20
19043 measured reflections	

### Refinement

**Table:**

Refinement on *F*^2^	Secondary atom site location: difference Fourier map
Least-squares matrix: full	Hydrogen site location: inferred from neighbouring sites
*R*[*F*^2^ > 2σ(*F*^2^)] = 0.024	H-atom parameters constrained
*wR*(*F*^2^) = 0.059	*w* = 1/[σ^2^(*F*_o_^2^) + (0.0273*P*)^2^ + 0.6672*P*] where *P* = (*F*_o_^2^ + 2*F*_c_^2^)/3
*S* = 1.07	(Δ/σ)_max_ = 0.002
4397 reflections	Δρ_max_ = 0.39 e Å^−^^3^
238 parameters	Δρ_min_ = −0.49 e Å^−^^3^
0 restraints	Extinction correction: *SHELXL97* (Sheldrick, 2008), Fc^*^=kFc[1+0.001xFc^2^λ^3^/sin(2θ)]^-1/4^
Primary atom site location: structure-invariant direct methods	Extinction coefficient: 0.00283 (17)

### Special details

**Table:**

Geometry. Bond distances, angles *etc*. have been calculated using the rounded fractional coordinates. All su's are estimated from the variances of the (full) variance-covariance matrix. The cell e.s.d.'s are taken into account in the estimation of distances, angles and torsion angles
Refinement. Refinement of *F*^2^ against ALL reflections. The weighted *R*-factor *wR* and goodness of fit *S* are based on *F*^2^, conventional *R*-factors *R* are based on *F*, with *F* set to zero for negative *F*^2^. The threshold expression of *F*^2^ > σ(*F*^2^) is used only for calculating *R*-factors(gt) *etc*. and is not relevant to the choice of reflections for refinement. *R*-factors based on *F*^2^ are statistically about twice as large as those based on *F*, and *R*- factors based on ALL data will be even larger.

### Fractional atomic coordinates and isotropic or equivalent isotropic displacement parameters (Å^2^)

**Table:**

	*x*	*y*	*z*	*U*_iso_*/*U*_eq_	Occ. (<1)
I1A	0.8199 (5)	0.0828 (2)	0.36895 (19)	0.0502 (6)	0.54 (4)
I1B	0.8299 (7)	0.0783 (3)	0.3658 (2)	0.0622 (8)	0.46 (4)
O1	0.6627 (3)	0.25061 (10)	0.31382 (12)	0.0512 (6)	
C1	0.9910 (4)	0.17057 (12)	0.15222 (17)	0.0408 (8)	
C2	0.9965 (5)	0.16572 (14)	0.06450 (19)	0.0573 (10)	
C3	0.8386 (6)	0.19473 (15)	0.01454 (19)	0.0637 (10)	
C4	0.6694 (6)	0.23029 (15)	0.05043 (19)	0.0621 (11)	
C5	0.6596 (4)	0.23677 (13)	0.13679 (17)	0.0473 (9)	
C6	0.8181 (3)	0.20662 (11)	0.18883 (15)	0.0350 (7)	
C7	0.8027 (3)	0.21515 (11)	0.28175 (15)	0.0356 (7)	
C8	0.9707 (4)	0.17888 (13)	0.33563 (17)	0.0439 (8)	
C9	1.1881 (4)	0.16933 (15)	0.2919 (2)	0.0546 (9)	
C10	1.1619 (4)	0.13632 (14)	0.20632 (19)	0.0518 (9)	
I2A	0.2833 (4)	0.51127 (14)	0.4019 (2)	0.0524 (4)	0.71 (3)
I2B	0.2948 (9)	0.5150 (3)	0.3949 (5)	0.0551 (13)	0.29 (3)
O2	0.1573 (3)	0.33920 (10)	0.34826 (14)	0.0590 (7)	
C11	0.4861 (4)	0.42382 (11)	0.19085 (17)	0.0395 (8)	
C12	0.4975 (5)	0.42831 (14)	0.1038 (2)	0.0593 (11)	
C13	0.3390 (7)	0.39932 (18)	0.0525 (2)	0.0735 (13)	
C14	0.1666 (6)	0.36502 (18)	0.0870 (2)	0.0725 (12)	
C15	0.1531 (4)	0.35857 (13)	0.1724 (2)	0.0528 (10)	
C16	0.3121 (3)	0.38740 (11)	0.22589 (16)	0.0366 (7)	
C17	0.2928 (4)	0.37754 (11)	0.31794 (16)	0.0385 (7)	
C18	0.4509 (4)	0.41620 (12)	0.37374 (17)	0.0431 (8)	
C19	0.6704 (4)	0.42740 (14)	0.33282 (19)	0.0504 (9)	
C20	0.6513 (4)	0.45891 (14)	0.24640 (19)	0.0496 (9)	
H2	1.11050	0.14215	0.03943	0.0687*	
H3	0.84562	0.19042	−0.04364	0.0763*	
H4	0.56189	0.24993	0.01653	0.0746*	
H5	0.54622	0.26144	0.16068	0.0568*	
H8	0.99519	0.20536	0.38710	0.0527*	
H9A	1.25810	0.21327	0.28537	0.0654*	
H9B	1.28268	0.14126	0.32686	0.0654*	
H10A	1.30098	0.13765	0.17757	0.0620*	
H10B	1.12210	0.08895	0.21380	0.0620*	
H12	0.61398	0.45126	0.07953	0.0712*	
H13	0.34957	0.40313	−0.00567	0.0882*	
H14	0.05908	0.34619	0.05236	0.0868*	
H15	0.03679	0.33471	0.19547	0.0632*	
H18	0.47290	0.39074	0.42614	0.0517*	
H19A	0.75860	0.45680	0.36852	0.0605*	
H19B	0.74537	0.38409	0.32833	0.0605*	
H20A	0.61044	0.50634	0.25223	0.0596*	
H20B	0.79302	0.45732	0.21957	0.0596*	

### Atomic displacement parameters (Å^2^)

**Table:**

	*U*^11^	*U*^22^	*U*^33^	*U*^12^	*U*^13^	*U*^23^
I1A	0.0533 (13)	0.0521 (9)	0.0453 (9)	−0.0069 (5)	0.0019 (5)	0.0139 (8)
I1B	0.084 (2)	0.0428 (9)	0.0599 (13)	−0.0067 (6)	0.0130 (10)	−0.0018 (11)
O1	0.0456 (10)	0.0531 (11)	0.0550 (12)	0.0073 (8)	0.0063 (8)	−0.0161 (9)
C1	0.0450 (12)	0.0273 (12)	0.0504 (16)	−0.0049 (9)	0.0138 (11)	0.0004 (11)
C2	0.0773 (19)	0.0391 (15)	0.0561 (19)	−0.0096 (13)	0.0319 (16)	−0.0049 (13)
C3	0.102 (2)	0.0490 (17)	0.0402 (16)	−0.0163 (17)	0.0091 (16)	0.0025 (14)
C4	0.083 (2)	0.0521 (18)	0.0510 (19)	−0.0057 (15)	−0.0130 (16)	0.0118 (14)
C5	0.0502 (14)	0.0387 (14)	0.0530 (17)	0.0023 (11)	−0.0036 (12)	0.0000 (12)
C6	0.0370 (11)	0.0243 (11)	0.0439 (14)	−0.0035 (9)	0.0054 (10)	−0.0011 (10)
C7	0.0322 (11)	0.0297 (12)	0.0448 (14)	−0.0045 (9)	0.0018 (10)	−0.0046 (10)
C8	0.0442 (13)	0.0389 (14)	0.0485 (15)	−0.0046 (10)	−0.0082 (11)	−0.0064 (11)
C9	0.0346 (12)	0.0499 (16)	0.079 (2)	−0.0019 (11)	−0.0078 (12)	0.0078 (15)
C10	0.0379 (13)	0.0419 (15)	0.076 (2)	0.0064 (10)	0.0160 (13)	0.0039 (14)
I2A	0.0475 (8)	0.0585 (9)	0.0512 (5)	0.0129 (3)	−0.0063 (3)	−0.0177 (5)
I2B	0.077 (3)	0.0322 (19)	0.0562 (13)	0.0131 (8)	0.0055 (13)	−0.0043 (12)
O2	0.0550 (11)	0.0514 (12)	0.0709 (14)	−0.0111 (9)	0.0206 (10)	0.0053 (10)
C11	0.0416 (12)	0.0274 (12)	0.0496 (16)	0.0011 (9)	0.0093 (11)	−0.0039 (10)
C12	0.0745 (19)	0.0488 (17)	0.0551 (19)	−0.0020 (14)	0.0204 (15)	0.0010 (14)
C13	0.105 (3)	0.071 (2)	0.0446 (18)	0.006 (2)	0.0004 (18)	−0.0078 (16)
C14	0.086 (2)	0.064 (2)	0.067 (2)	−0.0004 (18)	−0.0220 (19)	−0.0170 (17)
C15	0.0511 (15)	0.0381 (14)	0.069 (2)	−0.0081 (11)	−0.0073 (13)	−0.0075 (13)
C16	0.0355 (11)	0.0259 (12)	0.0484 (15)	0.0012 (9)	0.0025 (10)	−0.0033 (10)
C17	0.0356 (11)	0.0283 (12)	0.0517 (15)	0.0032 (9)	0.0071 (10)	0.0012 (10)
C18	0.0445 (13)	0.0427 (14)	0.0420 (15)	0.0102 (10)	−0.0023 (11)	0.0008 (11)
C19	0.0341 (12)	0.0504 (16)	0.0665 (19)	0.0033 (10)	−0.0068 (12)	−0.0087 (13)
C20	0.0347 (12)	0.0433 (15)	0.071 (2)	−0.0092 (10)	0.0103 (12)	−0.0039 (13)

### Geometric parameters (Å, °)

**Table:**

I1A—C8	2.170 (5)	C9—H9B	0.9700
I1B—C8	2.211 (6)	C10—H10A	0.9700
I2A—C18	2.180 (4)	C10—H10B	0.9700
I2B—C18	2.192 (7)	C11—C12	1.389 (4)
O1—C7	1.220 (3)	C11—C16	1.402 (3)
O2—C17	1.223 (3)	C11—C20	1.503 (4)
C1—C6	1.404 (3)	C12—C13	1.384 (5)
C1—C2	1.398 (4)	C13—C14	1.370 (5)
C1—C10	1.506 (4)	C14—C15	1.367 (5)
C2—C3	1.369 (5)	C15—C16	1.405 (4)
C3—C4	1.377 (5)	C16—C17	1.482 (4)
C4—C5	1.381 (4)	C17—C18	1.511 (4)
C5—C6	1.400 (4)	C18—C19	1.513 (4)
C6—C7	1.490 (4)	C19—C20	1.510 (4)
C7—C8	1.510 (4)	C12—H12	0.9300
C8—C9	1.518 (4)	C13—H13	0.9300
C9—C10	1.514 (4)	C14—H14	0.9300
C2—H2	0.9300	C15—H15	0.9300
C3—H3	0.9300	C18—H18	0.9800
C4—H4	0.9300	C19—H19A	0.9700
C5—H5	0.9300	C19—H19B	0.9700
C8—H8	0.9800	C20—H20A	0.9700
C9—H9A	0.9700	C20—H20B	0.9700
			
C2—C1—C6	118.2 (2)	C12—C11—C16	118.3 (2)
C2—C1—C10	121.1 (2)	C12—C11—C20	121.1 (2)
C6—C1—C10	120.7 (2)	C16—C11—C20	120.6 (2)
C1—C2—C3	121.8 (3)	C11—C12—C13	121.3 (3)
C2—C3—C4	120.0 (3)	C12—C13—C14	120.3 (3)
C3—C4—C5	119.9 (3)	C13—C14—C15	119.8 (3)
C4—C5—C6	120.8 (2)	C14—C15—C16	121.0 (3)
C1—C6—C5	119.3 (2)	C11—C16—C15	119.3 (2)
C1—C6—C7	121.5 (2)	C11—C16—C17	121.8 (2)
C5—C6—C7	119.21 (19)	C15—C16—C17	118.9 (2)
O1—C7—C6	122.0 (2)	O2—C17—C16	122.1 (2)
O1—C7—C8	120.6 (2)	O2—C17—C18	120.7 (2)
C6—C7—C8	117.38 (18)	C16—C17—C18	117.2 (2)
I1A—C8—C7	105.13 (17)	I2A—C18—C17	104.62 (17)
I1A—C8—C9	112.4 (2)	I2A—C18—C19	112.60 (18)
I1B—C8—C7	106.32 (18)	I2B—C18—C17	105.0 (2)
I1B—C8—C9	109.5 (2)	I2B—C18—C19	109.1 (2)
C7—C8—C9	113.1 (2)	C17—C18—C19	112.7 (2)
C8—C9—C10	112.3 (2)	C18—C19—C20	112.9 (2)
C1—C10—C9	112.9 (2)	C11—C20—C19	113.1 (2)
C1—C2—H2	119.00	C11—C12—H12	119.00
C3—C2—H2	119.00	C13—C12—H12	119.00
C2—C3—H3	120.00	C12—C13—H13	120.00
C4—C3—H3	120.00	C14—C13—H13	120.00
C3—C4—H4	120.00	C13—C14—H14	120.00
C5—C4—H4	120.00	C15—C14—H14	120.00
C4—C5—H5	120.00	C14—C15—H15	119.00
C6—C5—H5	120.00	C16—C15—H15	119.00
I1A—C8—H8	109.00	I2A—C18—H18	109.00
I1B—C8—H8	111.00	I2B—C18—H18	112.00
C7—C8—H8	109.00	C17—C18—H18	109.00
C9—C8—H8	109.00	C19—C18—H18	109.00
C8—C9—H9A	109.00	C18—C19—H19A	109.00
C8—C9—H9B	109.00	C18—C19—H19B	109.00
C10—C9—H9A	109.00	C20—C19—H19A	109.00
C10—C9—H9B	109.00	C20—C19—H19B	109.00
H9A—C9—H9B	108.00	H19A—C19—H19B	108.00
C1—C10—H10A	109.00	C11—C20—H20A	109.00
C1—C10—H10B	109.00	C11—C20—H20B	109.00
C9—C10—H10A	109.00	C19—C20—H20A	109.00
C9—C10—H10B	109.00	C19—C20—H20B	109.00
H10A—C10—H10B	108.00	H20A—C20—H20B	108.00
			
C6—C1—C2—C3	−0.3 (4)	C16—C11—C12—C13	1.7 (4)
C10—C1—C2—C3	177.9 (3)	C20—C11—C12—C13	−176.3 (3)
C2—C1—C6—C5	−0.6 (3)	C12—C11—C16—C15	−1.8 (3)
C2—C1—C6—C7	−178.9 (2)	C12—C11—C16—C17	177.3 (2)
C10—C1—C6—C5	−178.8 (2)	C20—C11—C16—C15	176.2 (2)
C10—C1—C6—C7	2.9 (3)	C20—C11—C16—C17	−4.7 (3)
C2—C1—C10—C9	156.0 (2)	C12—C11—C20—C19	−157.1 (2)
C6—C1—C10—C9	−25.8 (3)	C16—C11—C20—C19	25.0 (3)
C1—C2—C3—C4	0.5 (5)	C11—C12—C13—C14	−0.3 (5)
C2—C3—C4—C5	0.2 (5)	C12—C13—C14—C15	−1.0 (5)
C3—C4—C5—C6	−1.1 (4)	C13—C14—C15—C16	0.9 (5)
C4—C5—C6—C1	1.3 (4)	C14—C15—C16—C11	0.6 (4)
C4—C5—C6—C7	179.6 (2)	C14—C15—C16—C17	−178.6 (3)
C1—C6—C7—O1	174.4 (2)	C11—C16—C17—O2	−171.0 (2)
C1—C6—C7—C8	−4.7 (3)	C11—C16—C17—C18	8.1 (3)
C5—C6—C7—O1	−4.0 (3)	C15—C16—C17—O2	8.1 (3)
C5—C6—C7—C8	177.1 (2)	C15—C16—C17—C18	−172.9 (2)
O1—C7—C8—I1A	87.3 (2)	O2—C17—C18—I2A	−89.8 (2)
O1—C7—C8—C9	−149.8 (2)	O2—C17—C18—C19	147.6 (2)
C6—C7—C8—I1A	−93.7 (2)	C16—C17—C18—I2A	91.2 (2)
C6—C7—C8—C9	29.3 (3)	C16—C17—C18—C19	−31.5 (3)
I1A—C8—C9—C10	66.6 (3)	I2A—C18—C19—C20	−65.9 (3)
C7—C8—C9—C10	−52.2 (3)	C17—C18—C19—C20	52.1 (3)
C8—C9—C10—C1	50.1 (3)	C18—C19—C20—C11	−48.6 (3)

### Hydrogen-bond geometry (Å, °)

**Table:**

*D*—H···*A*	*D*—H	H···*A*	*D*···*A*	*D*—H···*A*
C5—H5···Cg3	0.93	2.95	3.700 (4)	139
